# Automatic detection on intracranial aneurysm from digital subtraction angiography with cascade convolutional neural networks

**DOI:** 10.1186/s12938-019-0726-2

**Published:** 2019-11-14

**Authors:** Haihan Duan, Yunzhi Huang, Lunxin Liu, Huming Dai, Liangyin Chen, Liangxue Zhou

**Affiliations:** 1 College of Computer Science, Sichuan University, South Section 1, Yihuan Road, Chengdu, 610065 Sichuan China; 2 College of Electrical Engineering, Sichuan University, South Section 1, Yihuan Road, Chengdu, 610065 Sichuan China; 3 Department of Biomedical Engineering, College of Materials Science and Engineering, Sichuan University, South Section 1, Yihuan Road, Chengdu, 610065 Sichuan China; 4 Department of Neurosurgery, West China Hospital, Sichuan University, No.37 Guo Xue Xiang, Chengdu, 610041 Sichuan China; 5 The Institute for Industrial Internet Research, Sichuan University, South Section 1, Yihuan Road, Chengdu, 610065 Sichuan China

**Keywords:** Intracranial aneurysm, Computer-aided diagnosis, Digital subtraction angiography, Object detection, Convolutional neural networks

## Abstract

**Background:**

An intracranial aneurysm is a cerebrovascular disorder that can result in various diseases. Clinically, diagnosis of an intracranial aneurysm utilizes digital subtraction angiography (DSA) modality as gold standard. The existing automatic computer-aided diagnosis (CAD) research studies with DSA modality were based on classical digital image processing (DIP) methods. However, the classical feature extraction methods were badly hampered by complex vascular distribution, and the sliding window methods were time-consuming during searching and feature extraction. Therefore, developing an accurate and efficient CAD method to detect intracranial aneurysms on DSA images is a meaningful task.

**Methods:**

In this study, we proposed a two-stage convolutional neural network (CNN) architecture to automatically detect intracranial aneurysms on 2D-DSA images. In region localization stage (RLS), our detection system can locate a specific region to reduce the interference of the other regions. Then, in aneurysm detection stage (ADS), the detector could combine the information of frontal and lateral angiographic view to identify intracranial aneurysms, with a false-positive suppression algorithm.

**Results:**

Our study was experimented on posterior communicating artery (PCoA) region of internal carotid artery (ICA). The data set contained 241 subjects for model training, and 40 prospectively collected subjects for testing. Compared with the classical DIP method which had an accuracy of 62.5% and an area under curve (AUC) of 0.69, the proposed architecture could achieve accuracy of 93.5% and the AUC of 0.942. In addition, the detection time cost of our method was about 0.569 s, which was one hundred times faster than the classical DIP method of 62.546 s.

**Conclusion:**

The results illustrated that our proposed two-stage CNN-based architecture was more accurate and faster compared with the existing research studies of classical DIP methods. Overall, our study is a demonstration that it is feasible to assist physicians to detect intracranial aneurysm on DSA images using CNN.

## Background

An intracranial aneurysm is a cerebrovascular disorder which is caused by localized dilation or ballooning of the internal carotid artery (ICA). An intracranial aneurysm appears to be associated with adult-dominant polycystic kidney disease (ADPKD), fibrous dysplasia, coarctation of the aorta, and so on [1]. Without timely detection and treatment, rupture of an intracranial aneurysm will lead to subarachnoid hemorrhage, which often results in serious neurological sequelae and has high fatality [2]. However, the traditional diagnostic approach is labor-consuming in that it requires the participation of skilled and experienced physicians. Nevertheless, failures to recognize intracranial aneurysms still happen from time to time. Therefore, developing an automatic system to assist doctors to accurately diagnose intracranial aneurysms can, in a certain degree, relieve the physicians’ burden.

Several research studies were conducted for automatically detecting intracranial aneurysms in recent years. The detection systems were tested on different angiographic modalities, such as magnetic resonance angiography (MRA) [3–7], and computed tomography angiography (CTA) [8–10]. Clinically, the invasive digital subtraction angiography (DSA) is taken as the gold standard of aneurysm detection instead of MRA and CTA for higher spatial resolution and sensitivity in the detection of small aneurysms [11]. Most existing computer-aided diagnosis (CAD) methods were based on classical digital image processing (DIP) methods using 2D-DSA images for some essential reasons. On one hand, with the invasive examination, DSA data are rather limited compared with non-invasive approaches (MRA or CTA). On the other hand, compared with the 2D-DSA modality, the 3D-DSA modality has more information that can easily identify aneurysms, but most hospitals in developing country can only afford 2D angiography devices for the expensive cost of 3D devices. However, current research studies based on classical DIP methods have some limitations. Abboud et al. [12] utilized morphology to predict the risk of rupture of an intracranial aneurysm by manual annotation, so their work lacks an automatic method to locate intracranial aneurysms. Rahmany et al. [13] fused a brief description of a priori knowledge from experts as fuzzy model to detect cerebral aneurysms. And then, Rahmany et al. [14] employed the Otsu method to extract the vascular structure and detect aneurysms with a combination of the Zernike moments and MSER detector. After that, Rahmany et al. [15] integrated MSER, SURF, and SIFT descriptors to do aneurysm detection, which could reduce the false positive rate compared with the previous work [14]. However, according to the results of above-mentioned studies, the classical DIP methods were not the best approach for feature extraction to represent the variety of aneurysms. And the sliding window approach which they applied was time-consuming during searching and feature extraction.

Different from classical DIP methods, convolutional neural network (CNN)-based methods [16] have been demonstrated to be more efficient in feature extraction. In recent years, the CNN architecture has been widely applied in object detection and obtained satisfactory response [17–22]. Moreover, the CNN architecture has also achieved good performance in several medical detection tasks [23–27]. Jerman et al. [28] computed intra-vascular distance to obtain an intensity map from 3D-DSA images, and used CNNs to classify whether the map represents an aneurysm. However, producing intensity maps takes a lot of computing time. Podgoršak et al. [29] modified VGG network to carry out a pixel-by-pixel semantic segmentation for vasculature and aneurysms, which achieved average AUC of 0.761. The 350 DSA acquisitions in their data set are completely comprised of saccular aneurysms, so the false-positive rate is not evaluated. Jin et al. [30] applied BiConvLSTM to identify aneurysms and achieved a sensitivity of 94.3%, with a high false-positive rate of 3.77 per sequence.

There are several challenges that remain to be solved in aneurysm detection using CNNs. First, the information loss of 2D-DSA images would increase the difficulty in diagnosing. Thus, it is imperative and meaningful to develop a method to remedy the information loss on 2D-DSA images. Second, DSA images with large field of view (FOV) and high resolution often contain unnecessary interference, which may introduce failures to identify tiny intracranial aneurysms and high computing time. Thirdly, the overlaps of vessels appear highly similar with intracranial aneurysms on DSA images. An efficient automatic detection system should avoid the misrecognition between the overlap and an aneurysm, since the false-positive rate is a significant clinic indicator. In this study, with 2D-DSA images, we proposed a CNN-based architecture to automatically detect intracranial aneurysms. The major contributions can be concluded as follows,

1. With our two-stage detection network, a step-wise localization can effectively reduce the interference from the background and promote the detection accuracy.
2. Using the information of both the frontal and lateral 2D-DSA sequences, the proposed automatic detection system can achieve state-of-the-art performance, with an effective false-positive suppression algorithm to better distinguish intracranial aneurysms from the overlaps of vessels.
3. The proposed architecture can notably increase the detection speed compared with classical DIP methods, with only millisecond reaction time.

## Results

### Region localization

**Fig. 1:**
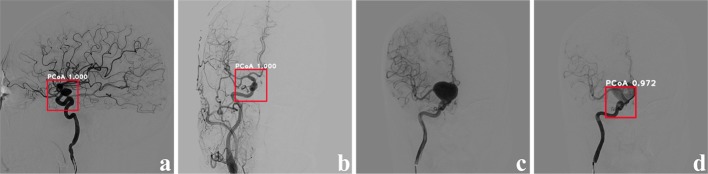
Example cases of RLS. In this figure, the red bounding box denoted PCoA region with the white annotation of region name and confidence. **a**, **b** Illustrated the PCoA region which could be detected accurately. **c** Was one of the images which did not find PCoA region. **d** Was another image from the same sequence of **c**, which could be detected with the PCoA region

The precision of RLS was 96%, in which the PCoA region was recognized from 96 images out of 100 testing images. Example instances are shown in Fig. 1. Figure 1a, b are cases that precisely located the PCoA region, with a confidence of 1.000. Fig. 1c is one of the images which did not find the PCoA region. Note that all pictures that did not successfully locate the PCoA region are from the same sequence which has an abnormally huge aneurysm. However, from the same sequence, Fig. 1d located the PCoA region with a confidence of 0.972. For the images that found the PCoA region, the IOU achieved 0.7441 with the *x*-axis and *y*-axis offset being 7.11% and 6.08%, respectively, which was a slight deviation that had a little impact for ADS.

### Comparison between dual input and single input

**Fig. 2:**
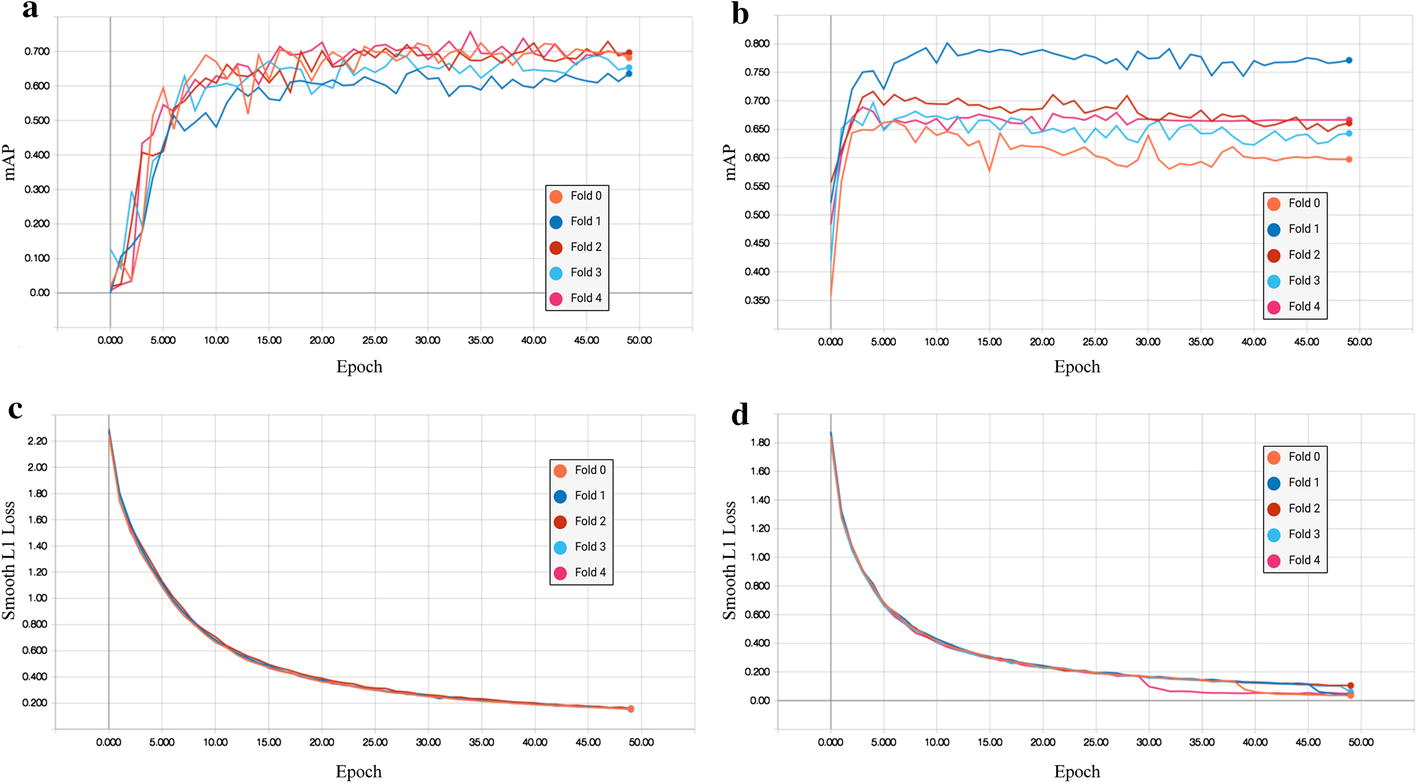
The fivefold cross-validation result curves of dual-input model and single-input model. Each curve of different colors denoted different cross-validation model. **a**, **b** Were mAP curves of dual-input model and single-input model, respectively. **c**, **d** Referred to Smooth L1 Loss curves of dual-input and single-input model, respectively

The result curves of fivefold cross validation of dual-input and single-input model are shown in Fig. 2, which features the mAP curves and Smooth L1 Loss curves. Figure 2a, b is the mAP curves of dual-input model and single-input model, respectively. In Fig. 2a, five curves of dual-input model are centralized and fluctuate between 0.6 and 0.7 after convergence. However, in Fig. 2b, the curves of single-input model are decentralized and distributed between 0.55 and 0.8 after convergence, with the highest bias between the fold 0 and fold 1 closed to 0.2. The result illustrates the dual-input model is more stable with different data compared with single-input model.

The Smooth L1 Loss was used to evaluate bounding-box regression accuracy. Figure 2c, d shows the curves of dual-input and single-input model, respectively. In Fig. 2d, the single-input model has some instability after 29 epochs. However, there is no obvious distance between these two models, because their losses are both under 0.2 after convergence which denotes that the result bounding box has only a slight deviation off manual annotations.

**Fig. 3:**
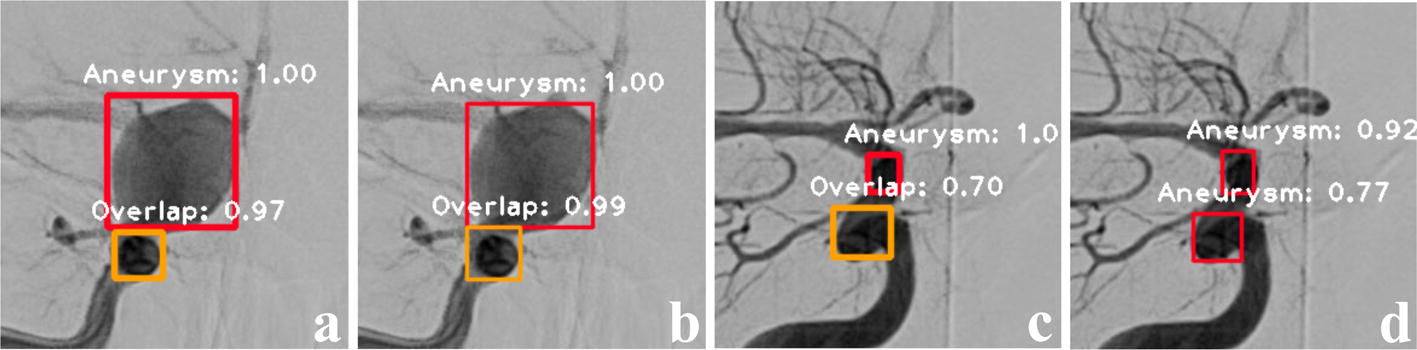
Example instances of ADS in PCoA region. In this figure, the red bounding box denoted aneurysm and the orange bounding box meant overlap of vessels, with the white annotation of class name and confidence. **a**–**d** Referred to the correct recognition results of dual-input model and single-input model respectively. Dual-input model could recognize the overlap in **c**, but single-input model misrecognized it in **d**

Example instances of ADS in PCoA region are displayed in Fig. 3. Figure 3a, b shows the outcome of dual-input model and single-input model, respectively, which are almost the same except the confidence of overlap. About Fig. 3c, d, the dual-input model that combines the frontal and lateral information can recognize the overlap with a confidence of 0.70, but the single-input model misrecognizes it as an aneurysm.

### Effect of regional average grayscale suppression

Clinical test could demonstrate the usability of RAGS algorithm. For comparison, we implemented the work based on the classical DIP method by Rahmany et al. [15] as the baseline of our study.

**Fig. 4:**
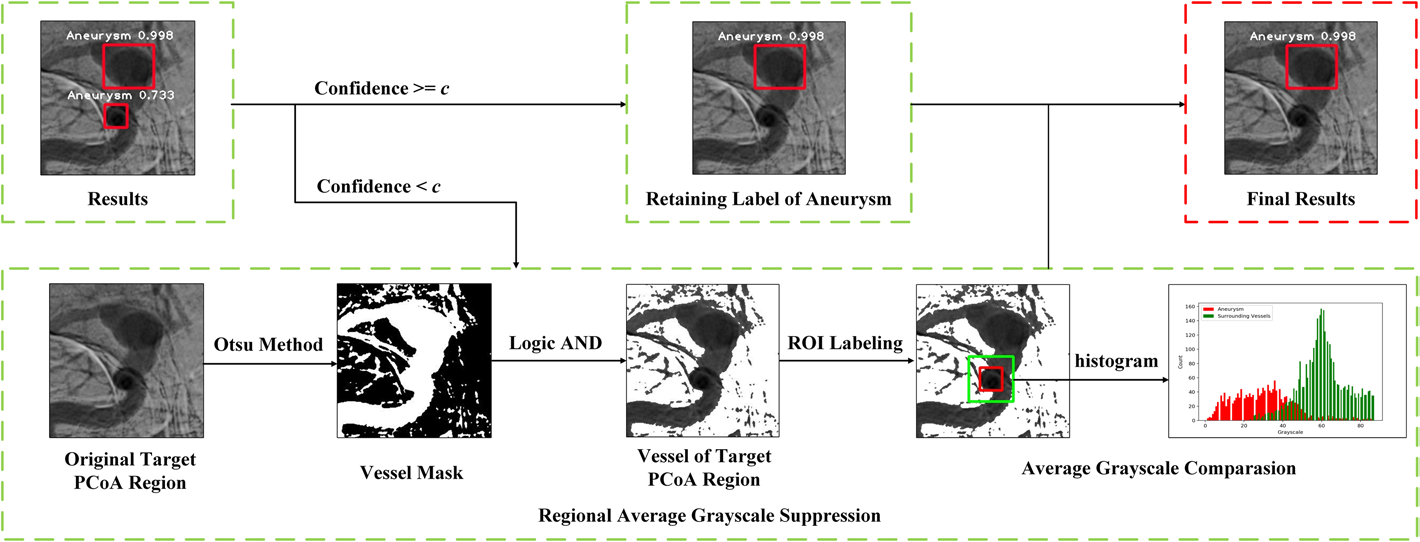
The flowchart of Regional Average Grayscale Suppression (RAGS) algorithm. In this figure, the red bounding box denoted aneurysm with the white annotation of region name and confidence in images of top raw. The pipeline of RAGS algorithm was displayed in bottom raw. In the rightest image, the red bounding box denoted ROI of aneurysm and the green bounding box denoted the ROI of enlargement area. And the histogram illustrated the difference of grayscale distribution between ROI of aneurysm (red bars) and ROI of enlargement area (green bars)

**Fig. 5:**
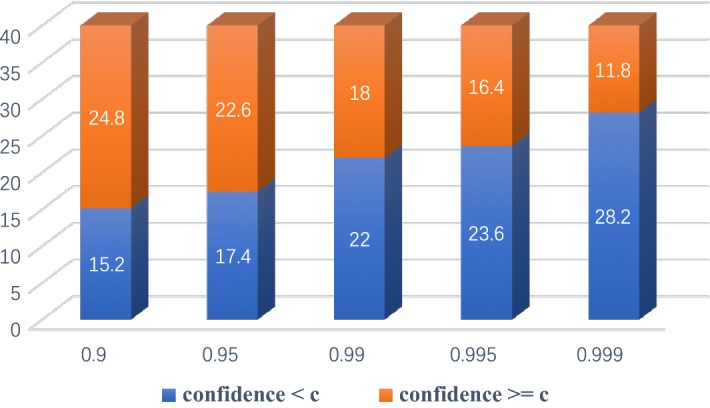
Distribution of patients for different choices of parameter *c*. The white annotations on bars were average count of patients. The orange part of bars denoted average count of patients whose disease confidence were equal or higher than *c*, and the blue part of bars meant average count of patients whose disease confidence was smaller than *c*

**Fig. 6:**
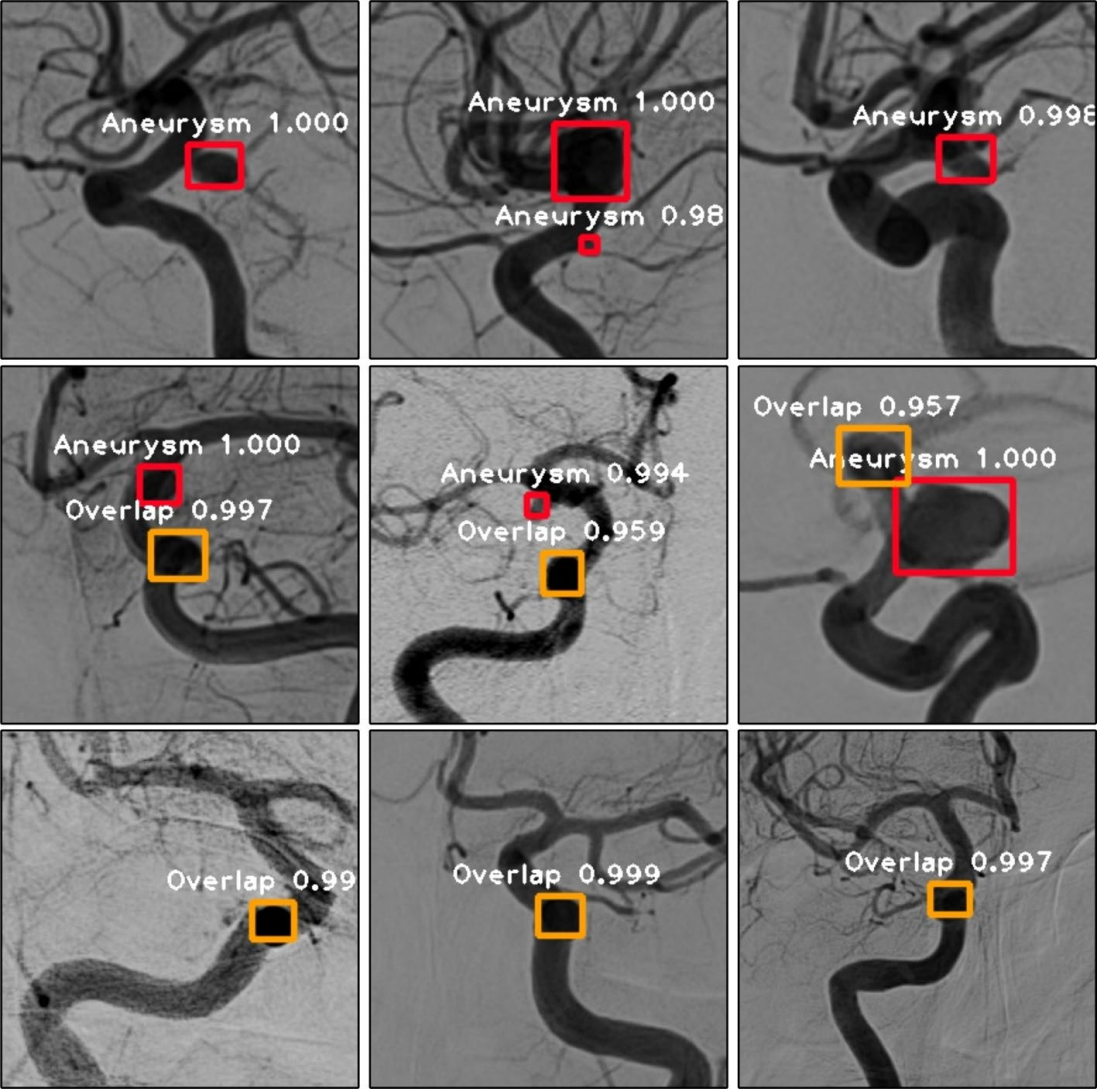
Sample results of proposed approach. In this figure, the red bounding box denoted aneurysm and the orange bounding box meant overlap of vessels, with the white annotation of class name and confidence

In previous section, we introduced parameter *c* as the threshold for RAGS algorithm. As shown in Fig. 4, if the confidence of a predicted aneurysm is lower than *c*, the RAGS algorithm will be applied. Otherwise, the label of aneurysm will be retained. Totally, 40 prospectively collected patients were involved for clinical tests, in which 20 patients received a positive diagnosis with an aneurysm in the PCoA region, while the other 20 patients received a negative result. We chose the highest confidence of a predicted aneurysm from the entire sequence of each patient as their disease confidence in our experiment. Figure 5 shows the average count distribution of patients for different choices of parameter *c*. To balance distribution, we manually picked 0.99 as parameter *c*. Sample results are illustrated in Fig. 6. Table 1 shows the confusion matrix of the clinical test. Table 1(a), (b) shows the confusion matrix of dual-input model with and without the RAGS, respectively. The dual-input model predicted 11 false-positive cases without RAGS, while there were only 1.8 false-positive cases and 0.8 false-negative cases after applying the RAGS algorithm. Table 1(c) shows the confusion matrix of the baseline method, which predicted 14 false-positive cases.

**Table 1:** Confusion matrix of clinical test

	Diagnosis+	Diagnosis−	Total
(a) Confusion matrix model of dual-input model (dual-input)			
Predict+	$20 {\pm } 0.00$	$11 {\pm } 2.97$	31
Predict−	$0 {\pm } 0.00$	$9 {\pm } 2.97$	9
Total	20	20	40
(b) Confusion matrix of dual-input model and RAGS (dual-input + RAGS)			
Predict+	$19.2 {\pm } 0.40$	$1.8 {\pm } 0.40$	21
Predict−	$0.8 {\pm } 0.40$	$18.2 {\pm } 0.40$	19
Total	20	20	40
(c) Confusion matrix of baseline method (Rahmany et al. [15])			
Predict+	19	14	33
Predict−	1	6	7
Total	20	20	40

**Fig. 7:**
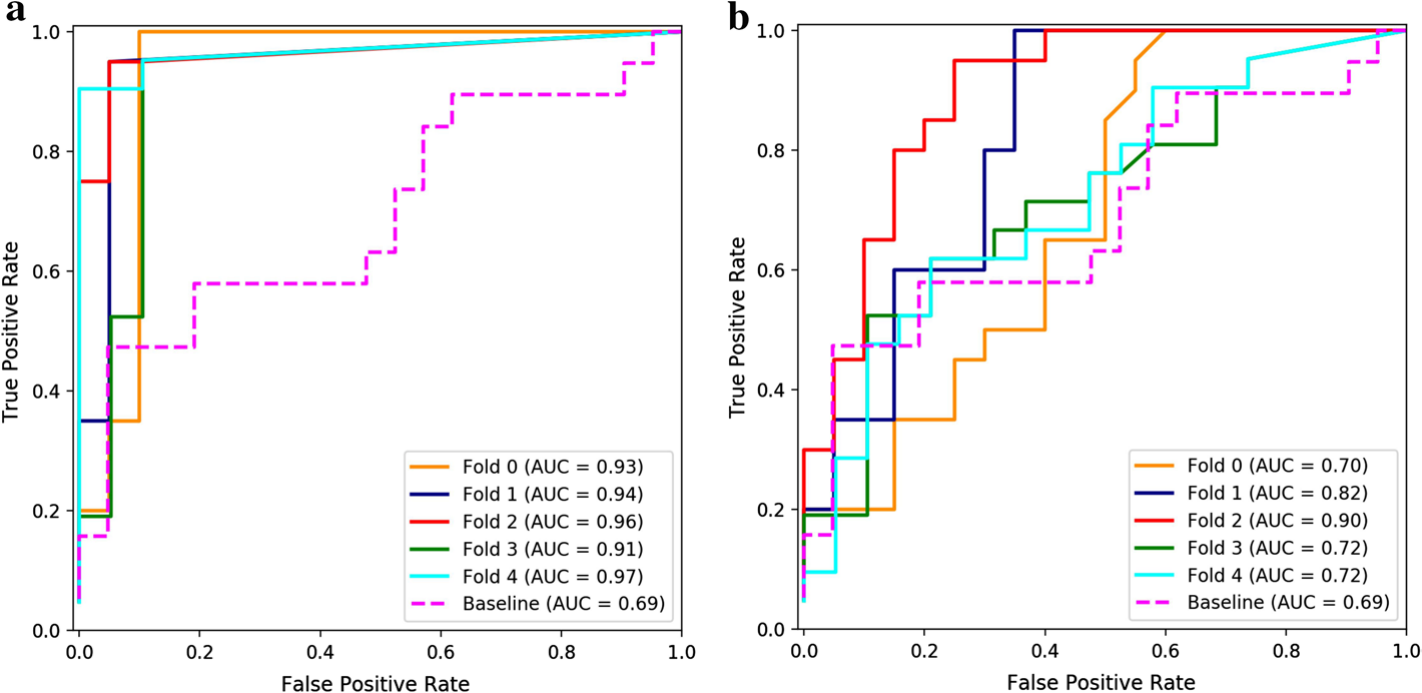
ROC curves of clinical test. **a**, **b** Was the ROC curve of the dual-input model with and without RAGS respectively. In this figure, each polyline of different colors denoted ROC curve of different cross-validation model of the proposed method, and the fuchsia broken line referred to ROC curve of baseline method

The ROC curves of clinical test are shown in Fig. 7. Therein, Fig. 7a, b shows the outcome of dual-input model with and without RAGS, where the average area under curves (AUC) is 0.942 and 0.772, respectively. As a contrast, the AUC of the baseline method was only 0.69.

Comparison of the performance for different methods is illustrated in Table 2. YOLOv3 [22], RetinaNet [31], and a physician with 20 years’ clinical experience were included as references. Note that the data which we utilized in model training were double checked by 3D-DSA modality to ensure the correctness of the annotations. However, the physician engaged in performance evaluation was only provided with the 2D images which were the input of the detector. As shown in Table 2, the best method among the six was the dual-input model with RAGS, which had sensitivity, specificity, and accuracy all higher than the physician. Although the dual-input model without RAGS and RetinaNet had a sensitivity of 1.000, their specificity was lower than 0.5. The method of Rahmany et al. had a sensitivity of 0.950, while the specificity and accuracy were only 0.300 and 0.625, respectively. With respect to the computation time cost, the CNN-based method was obviously faster than the classical DIP method. The quickest approach is YOLOv3 with only 0.0232s, and the proposed method took 0.0569s for diagnosing a patient, which was one hundred times faster than the baseline method approximately.

**Table 2:** Comparison of the performance for each method

	Sensitivity	Specificity	Accuracy	Time cost (s)
Dual-input	*1:000 (0:832; 1:000)*	0.450 (0.231, 0.685)	0.725 (0.561, 0.854)	0:0366 ± 0:0001
Dual-input + RAGS	0.960 (0.751, 0.999)	*0:910 (0:683; 0:988)*	*0:935 (0:796; 0:984)*	0:0569 ± 0:0013
Rahmany et al. [15]	0.950 (0.751, 0.999)	0.300 (0.119, 0.543)	0.625 (0.458, 0.773)	62:5460 ± 23:222
YOLOv3 [22]	0.850 (0.621, 0.968)	0.700 (0.457, 0.881)	0.775 (0.616, 0.892)	*0:0232 ± 0:0005*
RetinaNet [31]	*1:000 (0:832; 1:000)*	0.420 (0.191, 0.639)	0.710 (0.535, 0.834)	0:0456 ± 0:0036
Physician	0.900 (0.683, 0.988)	0.900 (0.683, 0.988)	0.900 (0.763, 0.972)	–

Data shown in “italic” are the highest value of the column

Data in parentheses are 95% CI

## Discussion

We examined the ability of our proposed CNN-based CAD architecture in region localization, aneurysm detection, and clinical test. The experimental results indicated that our two-stage framework was feasible for clinical application. For each stage of our experiment, the 96% precision of RLS demonstrated that our method could locate PCoA region for most patients. However, missing detection occurred on an angiographic sequence of a special patient. One of the unrecognized cases is shown in Fig. 1c, d is an image from the same sequence that accurately detected the PCoA region. Comparing the two images, we noticed that the case in Fig. 1c was very special, since the main vessel junction was occluded by a huge aneurysm. It seems that a necessary feature was lost for the neural network to locate the PCoA region because of the occlusion. This reveals that more special patients should be employed for model training to improve the robustness of RLS.

Combining the images of both the frontal and lateral view is helpful in clinical diagnosing, which inspired us to introduce the dual-input in ADS. As shown in Fig. 2a, b, the mAP curves of dual-input model were obviously more centralized after convergence comparing with the single-input model. On the other side, as shown in Fig. 3c, d, it was easier for the dual-input model to distinguish the overlap, while the single-input model misrecognized it as an aneurysm. Taken together, these results suggests that our proposed dual-input model has good stability and detection precision, since dual-input model can relatively remedy the information loss of single 2D-DSA image. Combining the images from another view, the CNN architecture can infer the vascular orientation, which is useful to distinguish the overlap of vessels.

Although the dual-input model decreased the false-positive cases compared with the single-input model, the false-positive rate was still exorbitant. In fact, some false-positive cases only had one or two frames diagnosed with an aneurysm in about 20 frames totally due to the strict evaluation indicator which we proposed for high sensitivity. However, this may point out that our evaluation indicator is not entirely reasonable, since the relationship between the sequence is not utilized.

The primary cause of false-positive cases is the misrecognition of the overlap of vessels. The results shown in Fig. 7 and Table 2 illustrate that the proposed RAGS algorithm could effectively improve the result, but it also led to a small set of false-negative cases. This might be because the intuition of RAGS algorithm is not completely reasonable, so that some exceptions can be misrecognized, such as low predicted confidence of large aneurysms or special viewing direction in angiography.

Besides, efficiency is an important indicator of CAD system. The speed of CNN-based methods was significantly faster compared with the classical DIP method, as shown in Table 2, since the sliding window method needs to traverse every window in DSA images to extract features, taking a lot of time. In contrast, CNN-based methods only need once forward propagation to extract feature map of the whole image, which effectively improved speed.

Unfortunately, several limitations of this work should be noted for further research. First, the data set is limited that it only contained 261 patients with aneurysms in PCoA region. Transfer learning method [32] can be applied if we can obtain data of other ICA regions in the future. In addition, DSA is an invasive examination modality, so that patients may not willing to choose. The wide accessible examination approaches such as MRA and CTA should be highly considered. Second, the relationship and information of each frame in an entire DSA sequence were not utilized. Recently, the recurrent neural network (RNN) has been reported for the ability of combining context information of a time series [16]. In future studies, we can apply RNN to deliver the features extracted by CNN for combining the context of DSA sequence. Third, the two-stage architecture might be a little complicated. For raw DSA images which have a resolution of $1024 \times 1024$, it is very difficult to search the tiny aneurysms in such high-resolution images. However, proposing an end-to-end detector beyond the limitation of a specific region is worthy of further research.

## Conclusion

In this study, we implemented a two-stage CNN-based architecture to detect intracranial aneurysms. A specific region would be located from original DSA images to reduce interference in RLS. Then, in ADS, intracranial aneurysms would be detected combining the information of frontal and lateral region image output from RLS, with RAGS algorithm for false-positive suppression. As shown in results, we demonstrated that our proposed CAD architecture was able to assist physicians to identify intracranial aneurysms efficiently.

## Methods

**Fig. 8:**
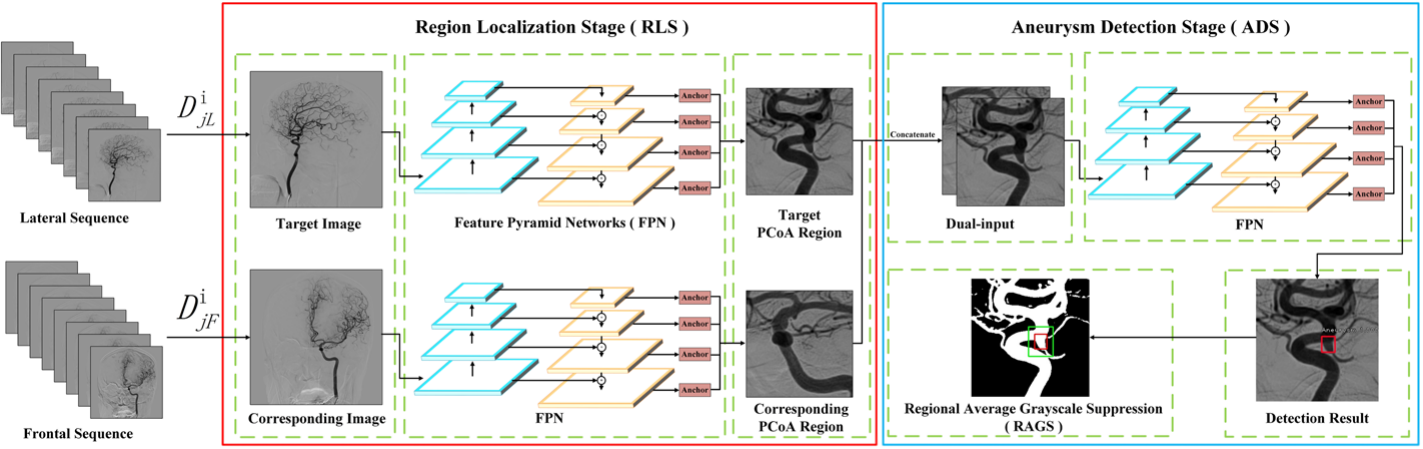
Schematic pipeline of our two-stage detection system. The region localization stage (RLS) located the PCoA region from the 2D-DSA images. The following aneurysm detection stage (ADS) identified intracranial aneurysm from the output of RLS

In this study, a two-stage CNN-based detection network was developed to implement the automatic detection of intracranial aneurysm on DSA images. The detection pipeline is schematized in Fig. 8. In region localization stage (RLS), our detection system paid attention to locating a specific region to reduce the interference from other regions. We experimented on posterior communicating artery (PCoA) region, which has the aneurysm recurrence rate of the second place among all ICA regions [33]. In aneurysm detection stage (ADS), intracranial aneurysms were identified on the output images from RLS. The CNN framework of above two stages were based on Feature Pyramid Networks (FPN) [31] with the backbone of ResNet50 [34]. The 2D-DSA device generated two matched sequences simultaneously for each patient, including the frontal and lateral view of head. Therefore, we introduced $D_{jF}^i$ to represent the *j*th image in frontal sequence of the *i*th patient and $D_{jL}^i$ for the corresponding lateral one. For example, as shown in Fig. 8, we used $D_{jL}^i$ to denote the target image which we want to make the detection, and $D_{jF}^i$ was its corresponding image which was at the same position from another angiographic view. In RLS, we input a target image and its corresponding image. Then, the model of RLS was applied to get the PCoA region images. In ADS, the dual-input concatenated the two PCoA region images, sending to the FPN of ADS to detect intracranial aneurysms. At last, we employed a region average grayscale suppression (RAGS) algorithm to suppress false-positive cases.

### Region localization stage

**Fig. 9:**
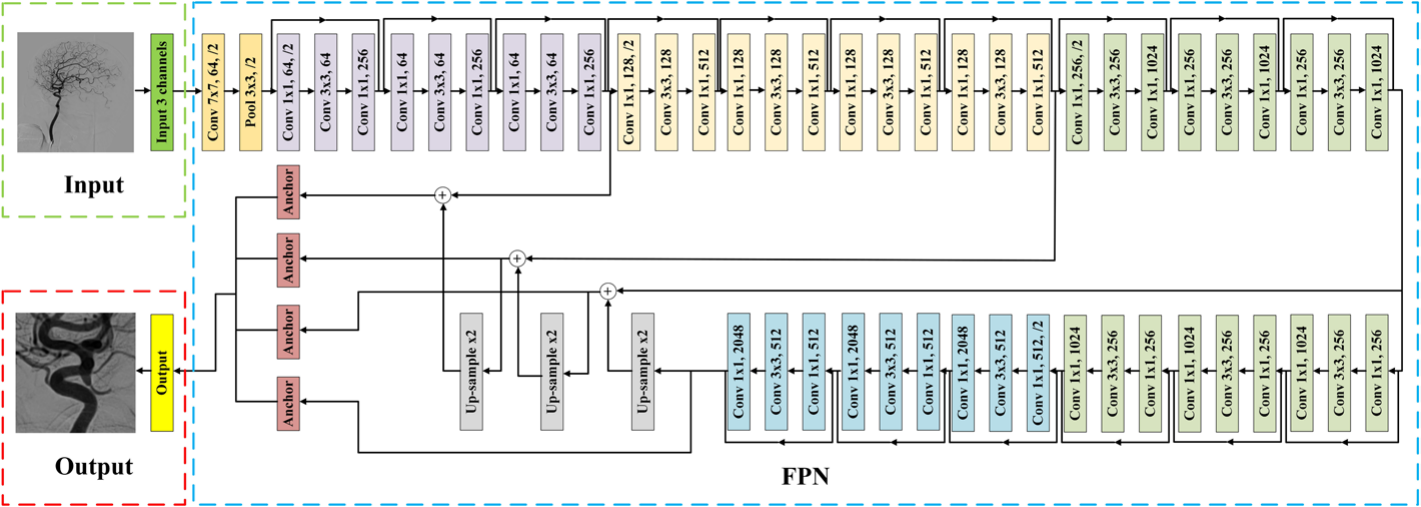
The architecture of region localization stage (RLS). In this figure, ‘${\text{Conv}} f{\times }f, c, /s$’ denoted convolutional layer with size of filters *f*, and number of channels *c* and strides *s* (default strides was 1). Noting that each Conv layer was followed by a BN and an activation layer of ReLU. ‘$\text{Pool} f {\times } f, /s$’ meant max pooling layer whose size of filters *f* and strides *s*. ‘${\text{Up-sample}}, /r$’ indicated nearest neighbor up-sampling with up-sampling rate *r*. ‘Anchor’ was anchor box which was utilized to predict the PCoA region. The first part was an input receiving a three-channel RGB image. The following feature extraction block was FPN with backbone of ResNet50. At last, the anchor boxes output the PCoA region of original input image

The large resolution of the raw 2D-DSA images can bring extra interference and time consumption, so it is imperative to mitigate the impact of unrelated tissues. We implemented the RLS to automatically locate a specific region, as shown in Fig. 9. To demonstrate the validity, we experimented on PCoA region.

The features of an input image was extracted by FPN, which could adapt various resolutions of input images and extract multi-scale features. The ResNet50 [34] was used as the backbone of FPN where each convolutional layer was followed by a batch normalization layer (BN) [35] and an activation layer of ReLU [36].

Then, the features were sent to anchor boxes [19] to predict the PCoA region of the original input image. In detail, the detector might predict 6*k* parameters totally. The feature was extracted by FPN, which was sensitive about various scales of object. The *k* stands for the number of objects, and the number 6 denotes one classification label, four parameters of bounding box (*x*, *y*, *w*, *h* denote *x*-coordinate, *y*-coordinate, width, and height, respectively) and one confidence for classification. Note that, using our architecture, other parts such as anterior communicating artery (ACoA) region can be conveniently extended with supplement of labeled data. At last, according to the result bounding box, our model output the image of PCoA region. Then, we resized the image to $288\times 288$ to unify the interface for easily transferring the detector of ADS to different ICA regions.

### Aneurysm detection stage

**Fig. 10:**
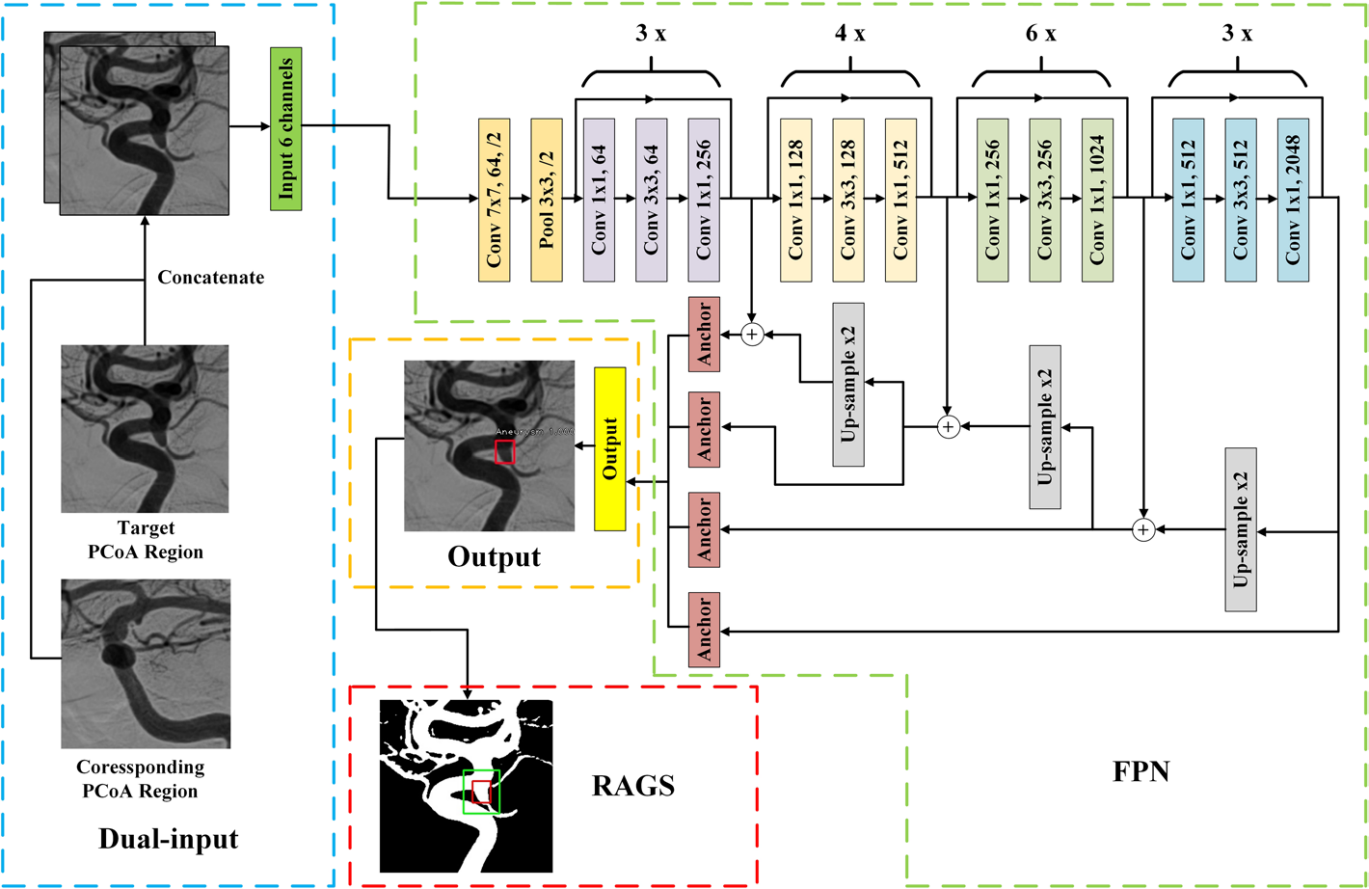
The architecture of aneurysm detection stage (ADS). The annotation of this figure was identical to Fig. 9. In addition, ‘${t}\times$’ on the top of brace denoted *t* times repeat of these layers. The dual-input layer concatenated the target PCoA region image and the corresponding PCoA region image, following with an FPN to cover various scales of aneurysms. Then, the anchor boxes output prediction results. The RAGS algorithm followed to suppress false-positive cases

The second stage of our pipeline was to distinguish between intracranial aneurysms and the overlaps of vessels. As illustrated in Fig. 10, our proposed architecture consisted of four steps, including: dual-input, feature extraction, output, and RAGS. The dual input concatenated the target PCoA region image and its corresponding PCoA region image as input tensor. The feature was extracted by FPN, which was sensitive about various scales of object. Then, the anchor boxes output the results of the detector. Similar to the RLS, the detector might predict 6*k* parameters totally, and the output step reserved the objects whose confidence was higher than 0.6. Finally, because the overlap of vessels influenced the detection result, the RAGS algorithm was applied on objects whose predicted label was aneurysm, but the confidence was lower than the pre-set threshold.

#### Dual input

Clinically, even experienced neurosurgeons may be confused in distinguishing intracranial aneurysms from the overlaps of vessels when providing either the frontal or lateral image. After providing both the frontal and the lateral DSA images, the experts can correctly infer the label through the trend of vessels. Motivated by this intuition, we modified the fundamental input architecture to combine the information of the frontal view and the lateral view. In details, the target PCoA image and the corresponding PCoA image output from RLS were concatenated as the input tensor of ADS. During concatenation, as shown in Fig. 10, we put the target image on the front with the corresponding one behind it. Therefore, the neural network can utilize the information from dual-input to partially remedy the information loss of 2D-DSA modality in feature extraction.

#### Regional average grayscale suppression

The dual-input model partly increased precision and stability, but the overlap of vessels was still easily misrecognized as an aneurysm in the preliminary experiment. Therefore, it was necessary to implement an algorithm to suppress the false-positive cases. According to the theory of DSA, the grayscale of image is determined by the density of radiocontrast agent [37]. Specifically, the grayscale of one pixel in 2D-DSA image depends on the density of radiocontrast agent in the blood within the pixel’s corresponding 3-D space. Therefore, the region of an overlap of vessels corresponds to more blood compared with surrounding region, which can result in deeper grayscale. The grayscale of very large aneurysms is usually deeper, because they contain lots of blood with radiocontrast agent, but they are easy to seek out by CAD method with an outstanding confidence closing to 1.0. On the contrast, for small aneurysms such as vessel umbos, the size of the aneurysms are usually smaller than the diameter of surrounding vein, so their grayscale would be lighter.

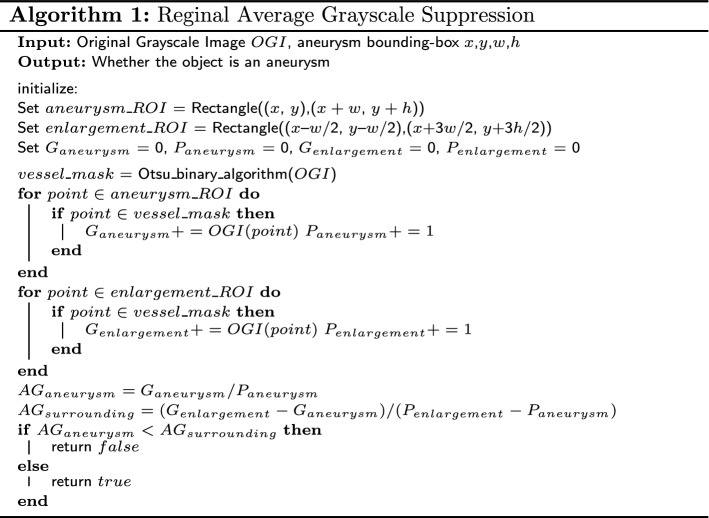



According to the principle mentioned above, we proposed RAGS algorithm to evaluate the output results which have the classification label of aneurysm with a confidence smaller than *c*. The flowchart is illustrated in Fig. 4 with pseudocode of Algorithm 1. We compared the confidence of predicted aneurysm with threshold *c* to determine whether the label of aneurysm would be retained. If the confidence of an aneurysm was lower than *c*, RAGS algorithm would be applied. At first, the vessel mask of original target PCoA region image was roughly extracted using the adaptive grayscale threshold selection method Otsu [38]. Then, the vessel of target PCoA region image was computed by logic AND operation using the vessel mask and original PCoA region image. In Fig. 4, the predicted bounding box of an aneurysm is displayed in red rectangle. To adapt different scales, we doubled the width and height at the same center of predicted aneurysm as the ROI of enlargement area, which is shown by green rectangle in Fig. 4. We also defined the region between green rectangle and red rectangle as ROI of surrounding vessels. After defining regions, we accumulated the gray values as $G_{\text{aneurysm}}$ and counted the pixels as $P_{\text{aneurysm}}$ of the aneurysm. So do an enlargement area as $G_{\text{enlargement}}$ and $P_{\text{enlargement}}$. The average grayscale of the aneurysm was counted as ${\text{AG}}_{\text{aneurysm}}$:

1 $$\begin{aligned} {\text{AG}}_{\text{aneurysm}}=G_{\text{aneurysm}}/P_{\text{aneurysm}}, \end{aligned}$$

and the average gray value of the surrounding vessels as ${\text{AG}}_{\text{surrounding}}$:

2 $$\begin{aligned} {\text{AG}}_{\text{surrounding}}=(G_{\text{enlargement}}-G_{\text{aneurysm}})/(P_{\text{enlargement}}-P_{\text{aneurysm}}). \end{aligned}$$

At last, comparing the ${\text{AG}}_{\text{aneurysm}}$ and ${\text{AG}}_{\text{surrounding}}$ was applied. If ${\text{AG}}_{\text{aneurysm}}$ is smaller than ${\text{AG}}_{\text{surrounding}}$, which denotes the grayscale of object is deeper than surrounding vessels, we discriminated that this object was an overlap. Otherwise, we retained its label of aneurysm.

### Data set

The data set was provided by Department of Neurosurgery, West China Hospital of Sichuan University, Chengdu, China. Since the radiocontrast agent needs to flow with blood, the visualization of intracranial arteries would vary with the position of radiocontrast agent. The experienced radiologists identified 6–12 frames for each patient when the radiocontrast agent flew through the intracranial arteries for sufficient visualization. Totally, 4976 images from 281 patients were collected, which contained both the frontal and lateral DSA sequences. The obtained images were in Digital Imaging and Communications in Medicine (DICOM) format from Philips FD20 Angio System which has a resolution of $1024 \times 1024$. The details of the data set are shown in Table 3. The aneurysms and overlaps of vessels were labeled by two experienced radiologists manually, which were double confirmed by 3D-DSA to ensure that the annotation procedure was correct. For a better usability, we converted the images to lossless Portable Network Graphics (PNG) format in model training and testing.

**Table 3:** Details of the data set

	Training dataset	Clinical test dataset
Gender		
Male	38 (15:77%)	3 (15:00%)
Female	203 (84:23%)	17 (85:00%)
Age		
$<18$	1 (0:41%)	0 (0:00%)
18–60	107 (44:40%)	9 (45:00%)
$>60$	133 (55:19%)	11 (55:00%)
Aneurysm dome size (mm)		
$<5.0$	42 (17:42%)	2 (10:00%)
5.0–9.9	106 (43:98%)	8 (40:00%)
10.0–24.9	92 (38:17%)	9 (45:00%)
$\ge 25.0$	1 (0:43%)	1 (5:00%)
Aneurysm neck size (mm)		
$<5.0$	122 (50:62%)	13 (65:00%)
$\ge 5.0$	119 (49:38%)	7 (35:00%)
Aneurysm dome size/aneurysm neck size		
$<1.5$	84 (34:85%)	7 (35:00%)
$\ge 1.5$	157 (65:15%)	13 (65:00%)
Aneurysm side		
Left	128 (53:11%)	12 (60:00%)
Right	113 (46:89%)	8 (40:00%)
Aneurysm status		
Rupture	93 (38:59%)	8 (40:00%)
Unrupture	148 (61:41%)	12 (60:00%)

The data set was partitioned into three portions. The first part was used for training of RLS. We randomly extracted 710 pictures of 36 subjects, which were manually annotated as the PCoA region for each picture by radiologists. There were up to 100 images from 5 randomly selected patients that were used for testing, and others for training. The training of ADS was fed with the second portion containing entirely 3786 images from 241 subjects, in which patients were diagnosed with an aneurysm in PCoA region. Particularly, this part of images were first processed by RLS. A clinical test after detector training utilized the third part including 40 prospectively collected patients, where 20 patients received a positive diagnosis with an aneurysm in PCoA region, while the other 20 patients received a negative result.

### Training process

In our experiment, the neural network was implemented using Keras [39], a deep learning framework using backend of Tensorflow [40]. The loss function for object classification used Focal Loss [31], defined as follows:

3 $$\begin{aligned} {FL}(p_t)=-{\alpha }(1-p_t)^\gamma \log (p_t), \end{aligned}$$

where $\alpha$ is a balanced parameter and $\gamma$ is down-weighted rate. We used $\alpha = 0.25$ and $\gamma = 2.0$ in the training process. The $p_t$ is defined as follows:

4 $$p_t= {\left\{ \begin{array}{ll} p & \quad \text {if} \,y=1 \\ 1-p & \quad \text {otherwise} \end{array}\right. }$$

where *p* denotes prediction confidence, and $y\in \{{\pm }1\}$ specifies the ground-truth class. For bounding-box regression, we used Smooth L1 Loss [31], defined as follows:

5 $$\begin{aligned} \text{SL}(t,v)=\sum _{i\in \{x,y,w,h\}}{\text{Smooth}}_{\text{L}}{1}(t_i-v_i), \end{aligned}$$

in which

6 $${\text{Smooth}}_{{\text{L}}1}(x)= {\left\{ \begin{array}{ll} 0.5({\sigma }x)^2 & \quad \text {if}\, |x| < 1\\ \frac{\left(|x|-0.5\right)}{\sigma ^2} & \quad \text {otherwise}, \end{array}\right. }$$

where *t* is the bounding box of the predicted object, *v* denotes the bounding box of ground truth. The $\sigma$ is a weighed factor, and we used $\sigma =3.0$ in experiment. The Adam Optimization method [41] was applied for training, with a learning rate of $\text{lr} = 3\times 10^{-6}$ for RLS, $\text{lr} = 10^{-5}$ for ADS, and two learning rate decay parameters of ${\beta }_1 = 0.9$, ${\beta }_2 = 0.999$. For a better initialization, the weights from ILSVRC [42] were transferred to be pre-trained model for faster convergence in fine-tuning process. The training process were performed on the platform with NVIDIA GTX 1080Ti GPU (11GB GDDR5X). The training process for RLS utilized 50 epochs and 1000 steps per epoch, which took 2 h. For ADS, the training process applied fivefold cross-validation scheme which used 50 epochs and 2500 steps per epoch, costing 7 h per model.

### Performance evaluation

The first metric of the RLS is precision. The precision can be defined as how many images the network can find the PCoA region from test data. Generally, detection task cares about the intersection over union (IOU). Slight movement or size variation of the bounding box is less influential for practical detection. Thus, a regularized offset to the centroid of manual annotation was proposed to evaluate the performance of RLS, defined as follows:

7 $$\begin{aligned} {\text{of}}\,f_x=\frac{|x_\text{annotation}-x_\text{prediction}|}{W} \end{aligned}$$

8 $$\begin{aligned} {\text{of}}\,f_y=\frac{|y_\text{annotation}-y_\text{prediction}|}{H}, \end{aligned}$$

where ${\text{of}}\,f_x$ means regularized offset of *x*-axis. $x_{\text{annotation}}$ is the *x* coordinate of centroid of manual annotation, and $x_{\text{prediction}}$ is the *x* coordinate of centroid of predication of the RLS. The same as the *y*-axis. The *W* and *H* denote width and height of manual bounding box, respectively.

Besides, a fivefold cross-validation scheme was applied in ADS. To compare the performance, five models of dual-input and single-input model were trained out using the same data set. The mean average precision (mAP) was employed to evaluate the classification precision of the aneurysm and overlap. For bounding-box regression task, we paid attention to the Smooth L1 Loss.

Moreover, the clinical purpose of our detection system is to give physicians an accurate hint that whether the patient has an aneurysm or not. To avoid missing diagnosis, a strict evaluation indicator was defined as follows:

9 $$\begin{aligned} \text{Diag}^i=\text{Diag}^i_{0F}{\vee }\text{Diag}^i_{1F}{\vee }{\ldots }{\vee }\text{Diag}^i_{(n-2)F}{\vee }\text{Diag}^i_{(n-1)F} \\ {\vee }\text{Diag}^i_{0L}{\vee }\text{Diag}^i_{1L}{\vee }{\ldots }{\vee }\text{Diag}^i_{(n-2)L}{\vee }\text{Diag}^i_{(n-1)L}, \end{aligned}$$

where $Diag^i$ means whether the *i*th patient has an aneurysm, $Diag_{jV}^i$ equals to true when the *j*th image from the *i*th patient on *V* perspective (*F* or* L*) was predicted with an aneurysm. In other words, this formula denotes that a patient will be defined with an aneurysm just if one frontal or lateral frame is detected with aneurysm among entire DSA sequence. Besides, the confusion matrix and receiver-operating characteristic (ROC) curve were employed in evaluation. And we counted the true positive (TP), false positive (FP), true negative (TN), and false negative (FN) to calculate sensitivity, specificity, and accuracy, which is defined as follows:

10 $$\begin{aligned} \text{Sensitivity} = {\text{True}} \; {\text{Positive}} \; {\text{Rate}} \, (\text{TPR})=\frac{\text{TP}}{\text{TP}+\text{FN}} \end{aligned}$$

11 $$\begin{aligned} \text{Specificity} = \text{True}\; \text{Negative} \; \text{Rate}\,(\text{TNR})=\frac{\text{TN}}{\text{FP}+\text{TN}} \end{aligned}$$

12 $$\begin{aligned} \text{Accuracy} = \frac{\text{TP}+\text{TN}}{\text{TP}+\text{FP}+\text{TN}+\text{FN}}. \end{aligned}$$

In addition, 95% confidence interval (CI) was evaluated, and computing time was calculated to estimate the efficiency of the CAD system.

## Data Availability

The data sets used and analyzed during the current study are available from the corresponding author on reasonable request.

## Abbreviations

DSA: digital subtraction angiography
CAD: computer-aided diagnosis
DIP: digital image processing
CNN: convolutional neural network
RLS: region localization stage
ADS: aneurysm detection stage
PCoA: posterior communicating artery
ICA: internal carotid artery
DICOM: Digital Imaging and Communications in Medicine
AUC: area under curve
CI: confidence interval
PNG: Portable Network Graphics
ADPKD: adult-dominant polycystic kidney disease
MRA: magnetic resonance angiography
CTA: computed tomography angiography
FOV: field of view
FPN: feature pyramid networks
RAGS: region average grayscale suppression
BN: batch normalization
ACoA: anterior communicating artery
ROI: region of interest
IOU: intersection over union
mAP: mean average precision
ROC: receiver-operating characteristic
TP: true positive
FP: false positive
TN: true negative
FN: false negative
RNN: recurrent neural network
